# Understanding the dynamics of *Meloidogyne incognita* infestation in pomegranate orchards of Himachal Pradesh, India (year 2018, 2019 and 2021) and its management strategies

**DOI:** 10.1016/j.heliyon.2024.e34752

**Published:** 2024-07-26

**Authors:** Mukesh, Kishore Khosla, Satish K. Sharma

**Affiliations:** a SRF, Department of Plant Pathology, Dr YS Parmar University of Horticulture and Forestry, Nauni, Solan, Himachal Pradesh, 173230, India; b Retired Professor, Plant Pathology, College of Horticulture and Forestry Thunag, Mandi, Dr YS Parmar University of Horticulture and Forestry Nauni, Solan, Himachal Pradesh, 173230, India; c Professor and Head, Department of Plant Pathology, Dr YS Parmar University of Horticulture and Forestry Nauni, Solan, Himachal Pradesh, 173230, India

**Keywords:** Pomegranate, Root knot-Nematode, *Meloidogyne incognita* and second stage-juvenile

## Abstract

This study investigates the prevalence and dynamics of pomegranate wilt disease induced by *Meloidogyne incognita* across the Kullu, Mandi and Solan districts of Himachal Pradesh (India), revealed notable spatial and temporal variations in nematode populations and galling severity across the regions. The highest average nematode infestation of 9.25 % was observed at Nauni with highest counts of average second-stage juvenile (J2) larvae (449 larvae per 100 cc of soil) followed by Hurla (Kullu) with 7.42 % infestation. Correlation analysis reveals a strong positive relationship between larval population and galling severity suggesting a potential link between nematode levels and plant damage. Common disease symptoms were leaf size reduction, yellowing and gradual decline of pomegranate plants, often observed in patches within orchards. Microscopic identification revealed distinctive pear-shaped body of mature females while J2 larvae displayed vermiform shapes and the associated species of *M. incognita* was confirmed through examination of the perineal pattern. Pathogenicity test reveals initiation of leaf yellowing symptom after 45 days of inoculation of larval suspension and root galling was observed after 60 days onward followed by plant decline under greenhouse conditions. Results from pot and field experiments demonstrated the efficacy of Fluopyram and Fluensulfone in reducing nematode populations and galling severity. Treatment with drenching of Fluopyram at the rate of 2 ml/L reduced 98.56 % larvae under field and 99.00 % larvae/100 cc soil under pot conditions. Statistical analysis (paired *t*-test and MANOVA) confirms significant differences in galling severity and larval population before and after drenching. The study also underscores the importance of weed management in disease mitigation as several weed species (*Chenopodium album* and *Solanum nigrum)* were identified as potential reservoirs for *M. incognita* in infested pomegranate plant basin. This investigation contributes to the advancement of management practices for pomegranate cultivation that addresses both nematode and weed infestations ultimately enhancing crop resilience and productivity.

## 1 Introduction

Pomegranate (*Punica granatum* L.) plant belonging to the Punicaceae family, a fruit-bearing deciduous shrub or small tree with multi-stems that can reach a height of 5–8m and has been cultivated since around 2000 BCE, primarily in Iran. Its importance has been steadily increasing due to its substantial nutritional and therapeutic value, resulting in a high demand in the global market [1]. The fruits are rich in carbohydrates and minerals such as calcium, iron and sulfur. Approximately half of the fruit is edible, composed mostly of water (85 %) along with sugar, organic acids, phenolics, flavonoids, polyphenols and isoflavones [2]. The fruit juice contains high levels of polyphenols, polysaccharides, lipids and secondary metabolites which are beneficial as antioxidents. The seeds of wild pomegranates, along with their fleshy portions, are dried and sold as "Anardana," a condiment used in Indian curries. Ideal conditions for pomegranate cultivation include arid and semi-arid regions with annual rainfall of 500–1000 mm with hot and dry summers and mild winters [3] In India, its cultivation covers an area of 2,34,000 ha, yielding approximately 2,845,000 metric tons of fruit and Maharashtra is considered the pomegranate basket of India, accounting for 54.85 % of the country's total production, followed by Gujarat [4]. In Himachal Pradesh, pomegranate is cultivated in subtropical conditions, covering an area of 2880 ha with a production of 3271 metric tons [5]. Its cultivation serve as a profitable alternative for farmers when apple and stone fruits are not performing well, offering potential income opportunities. The Kullu valley in Himachal Pradesh has gained popularity for its quality pomegranate production, attributed to lower monsoon rainfall compared to other parts of the state. However, there is still scope for expanding pomegranate cultivation in other subtropical regions of the state, with the adoption of advanced production and water harvesting technologies to increase productivity and its significant export potential.

Pomegranate wilt has been reported in several countries around the world, including China [6] Oman [7] Pakistan [8] and India [9]. Various genera of nematodes, including *Meloidogyne* sp., *Pratylenchus* sp., *Paratrichodorus* sp., *Helicotylenchus* sp., *Rotylenchulus* sp., *Tylenchorhynchus* sp., *Longidorus* sp., *Xiphinema* sp. and *Criconemella* sp. have been reported in pomegranate orchards [[10], [11], [12], [13]]. Among these nematodes, root knot nematodes pose a greater challenge due to their diverse range of hosts, short life span and adaptability to various environmental conditions [14,15]. Root knot nematodes belong to the domain Eukaryota, kingdom Metazoa, phylum Nematoda, class Secernentea, order Tylenchida, family Heteroderidae, genus Meloidogyne and species *M. incognita* [16]. Each species of Meloidogyne can be identified by its unique posterior cuticular pattern [17]. The life cycle of root knot nematodes begins with eggs in the soil, which develop into first-stage juvenile (J1) nematodes which molt into second-stage juveniles (J2) are motile and parasitize plant cells, transforming them into giant cells for feeding. It penetrates the roots of susceptible plants that cause the formation of knots or galls which disrupt nutrient and water uptake system that induces yellowing and partial wilting symptoms on infested pomegranate plant [18]. The transition from juvenile to vermiform adult males, globose females occurs at the J4 stage and a single female can produce 500–1000 eggs [19]. The first observation of *M. incognita* symptoms, such as pinhead excrescences on cucumber plant roots, was made by Berkeley in 1855 in Nuneham, England [20]. Root knot nematodes infect a wide range of fruit crops including apple, peach, plum, cherry, walnut, pomegranate, strawberries, grapes, citrus fruits, bananas, peaches, apricots, plums [21] and vegetable crops of the Cucurbitaceae, Solanaceae, Umbelliferae and Brassicaceae families [22,23]. *M. incognita* also infest several weed hosts i.e. *Amaranthus blitoides*, *Portulaca oleracea*, *Polygonum aviculare*, *Convolvulus arvensis*, *Cyperus rotundus*, *Plantago lanceolatum*, *Rumex acetosa*, *Solanum nigrum*, *Datura stramonium*, *Acroptilon repens*, *Alcea rosa*, *Alhaji camelorum*, *Chenopodium album*, *Echinochla crusgalli*, *Hibiscus trionum*, *Kochia scoparia*, *Malva rotundifolia*, *Setaria viridis* and *Lactuca serriola* with varying rates of egg mass production and galling on roots [24]. Leaf wilting and yellowing due to nematode infestation have profound economic repercussions on orchard productivity can directly affect the health and yield potential of pomegranate trees, leading to reduced fruit quality and quantity, increased production costs and potential long-term declines in orchard sustainability. To mitigate these impacts, early detection and effective nematode infestation management strategies are essential. Implementing regular monitoring programs, conducting soil tests for nematode population levels and utilizing nematicides drenching judiciously are crucial steps in minimizing nematode damage and maintaining orchard productivity. Effective management of pomegranate wilt disease requires an integrated approach, including cultural operations like proper drainage systems, weed management and the use of chemical nematicides. Chemical treatments have proven to be reliable and useful for managing ongoing infestation under field conditions that reduces larval population in soil [25].

Root-knot nematode infestation pose a significant risk to pomegranate farming in Kullu Mandi and Solan districts resulting in reduced yields and financial strains of the growers. The intricate nature of pomegranate wilt disease, coupled with the presence of other fungal plant pathogens (*Ceratocystis fimbriata*, *Fusarium oxysporum* and *Rhizoctonia* sp.) with limited understanding of specific nematode infestations, presents a formidable challenge in selecting appropriate chemical groups for effective disease management [26]. This underscores the imperative for thorough investigation to develop comprehensive strategies addressing the diverse pathogens affecting pomegranate cultivation. Therefore present investigation monitored the prevalence and infestation of *M. incognita* in pomegranate orchards across Kullu, Mandi and Solan districts of Himachal Pradesh during year 2018, 2019 and 2021 then proved its pathogenic nature through pot experiments. This study also recorded the types of weeds growing around infested pomegranate tree basin and association of *M. incognita* with the weed plant during the surveillance program. Fluopyram (34.48 % SC) and flunosulfone (2 % GR) were evaluated under both pot and field conditions to assess their efficacy in reducing larval populations and galling index in infested soil and plant roots. These chemical nematicides have the potential to induce paralysis and eventual death of *M. incognita* larvae under field conditions. The novelty of the study lies in systematically monitoring nematode dynamics in Himachal Pradesh over multiple years and locations which provides a comprehensive understanding of infestation patterns. Additionally, the identification of weed species associated with nematode infestations highlights potential alternative hosts contributing to nematode proliferation. The study introduces a novel management strategy that combines weed management with chemical treatments, aiming to reduce nematode inoculum in the soil. This integrated approach minimizes nematode populations in soil, improve soil health that helps in reducing yield losses and also ensure the sustainable long-term cultivation of pomegranates worldwide.

## 2 Future Perspectives of the current research work

The current research establishes a strong basis for future advancements offering several avenues to enhance pomegranate yield and quality while mitigating the impact of root knot nematodes. Incorporating precision agriculture technologies i.e. remote sensing, GIS mapping and automated monitoring systems can significantly improve the accuracy of nematode detection. Developing comprehensive integrated pest management (IPM) strategies that combine cultural practices, biological controls and selective chemical treatments can offer a holistic approach to managing nematode infestations. Employing botanicals and beneficial microbes (mycorrhizal fungi, rhizobacteria and nematode-trapping fungi) can offer eco-friendly alternatives to chemical nematicides enhancing soil health and providing sustainable pest management solutions. Molecular biology and genetic engineering techniques can be utilized to identify and develop nematode-resistant pomegranate cultivars potentially revolutionizing disease management. The integration of advanced scientific techniques, sustainable practices and farmer-centric approaches will contribute significantly to the global pomegranate industry, enhancing food security and economic prosperity.

## 3 Material and methods

The present investigation was carried out in Department of Plant Pathology, Dr YS Parmar University of Horticulture and Forestry Nauni, Solan (HP) India.

### 3.1 Surveillance

Comprehensive surveillance of major pomegranate growing areas in the Kullu, Mandi and Solan districts of Himachal Pradesh (India) were conducted during year 2018, 2019 and 2021 to assess the occurrence of disease in pomegranate orchards by visually observing symptoms like yellowing, wilting and formation of galls on roots. The survey followed a systematic approach, where 4 locations were selected each district and at each locations, three orchards were randomly chosen for observation. In each orchard, a total of 60 plants were examined to assess the percentage of disease incidence calculated by using formula given below.

$$\text{Disease}\;\text{incidence}\;\left(\%\right)=\frac{\text{Number}\;\text{of}\;\text{diseased}\;\text{plants}}{\text{Total}\;\text{number}\;\text{of}\;\text{plants}\;\text{observed}}\times 100$$

The root system of each nematode infested plant was rated for galling index (GI) by using 0 to 5 scale [27] where, 0 = no galls, 1 = 1–2 galls; 2 = 3–10 galls; 3 = 11–30 galls; 4 = 31–100 galls and 5 = > 100 galls per root system. During the field surveys the composite samples of soil and roots with galls were collected from infected pomegranate plants. The soil sampling was done from four sides of the plant approximately 45–60 cm away from the stem at a depth of 20–40 cm in the rhizosphere [28] and randomly five infested pomegranate plants were selected for sampling from which 200 g of composite soil samples were taken out from every location to the laboratory for processing and calculating the distribution of J2 nematodes in the soil.

### 3.2 Isolation and identification of root knot nematode

In the Plant Nematology Laboratory at Department of Entomology the juvenile larvae were extracted from the soil using two techniques: Cobb's decanting & sieving method and the modified Baermann's funnel method [29]. 100 cc soil for each samples taken during surveillance programme taken, mixed in 1 L water and the suspension was then passed through series of 20, 60 and 300mesh sieve under tap water. The nematode suspension from 300 mesh sieve were collected in a separate beaker and then poured in a petriplate through wire net covered with double layer tissue paper. The samples were scrutinized to access the number of second-stage juvenile (J2) of *M. incognita* present by placing 1 ml of the nematode suspension onto a counting dish and calculating sum of the J2 larval present in each square under stereoscopic microscope. Mature female of *M. incognita* was extracted dissecting the knot produced on the roots and identification of the *Meloidogyne* species was performed by observing the distinctive perineal pattern by cutting the posterior end of the female nematode, mounting on a drop of anhydrous glycerin on a glass slide covered with a cover slip and viewed at 40× magnification using microscope [30]. Measurements of the total body length (μm), maximum body width (μm) and stylet length (μm) of both second-stage juvenile larvae and mature females were recorded under the microscope and identification was performed by comparing these measurements with data gathered from previous studies on *M. incognita*. Combining traditional morphological examination offers a robust framework for the comprehensive identification of nematode species infecting pomegranate roots. It infection can easily identified in a orchard by symptoms like wilting of infested plants and presence of knots on roots under field conditions. The morphological features of juvenile and adult nematodes added reliability and the validation of nematode virulence through pot-based pathogenicity assays solidifies the methodology's efficacy in assessing the impact of nematode infestation on pomegranate health. This multidisciplinary approach lays the groundwork for implementing targeted management strategies to mitigate the detrimental effects of nematode infections in pomegranate cultivation.

### 3.3 Pot evaluation

#### 3.3.1 Pathogenicity test

Pathogenicity test was carried on pomegranate seedling (Kandhari Kabuli) grown on plastic pots (45 × 25 cm size) maintained prior to the study in the greenhouse located at the Department of Plant Pathology.

##### 3.3.1.1 Collection of egg masses of nematodes

Infested pomegranate plant roots affected by *M. incognita* were collected, washed and rinsed in water for a period of 1–2 h. Subsequently, the egg masses present on the roots were collected in a petri plate containing water which is placed in an incubator set at a temperature of 28 °C for duration of 48 h, allowing the second-stage juveniles to hatch from the eggs. After the incubation period, the suspension containing the hatched larvae were examined under a stereoscopic binocular microscope and counted by using the previously described method.

##### 3.3.1.2 Inoculation of nematode suspension culture

A 10 ml suspension containing 1000 nematode larvae per plant were poured twice at 15 days interval around the roots of a potted pomegranate plant. The inoculated plants were then monitored for the disease symptoms and the number of days taken to induce first symptom was recorded. The feeder roots of the infested plants in the pots were regularly examined for number of days taken to formation of galls or knots and the mature females of *M. incognita* extracted from well developed galls on the roots. The associated species was confirmed by examining the posterior cuticular pattern of female *M. incognita* extracted from newly infested plants using the stereoscopic microscope by using previously described methods.

### 3.4 Management of pomegranate wilt disease

#### 3.4.1 Cultural practices

Cultural practices are pivotal in addressing pomegranate wilt particularly in orchards facing challenges like inadequate drainage or environmental stresses such as drought or excessive moisture. In orchards with poor drainage facility, 15–30 cm deep and 10–20 cm wide trenches were excavated between plant rows based on the area's topography to enhance soil drainage an mitigate the disease under field condition. Furthermore, the presence of weed species within the infested plant basins was documented and monitored for root-knot nematode (RKN) infection. As part of the management approach, all weeds in the infested plant basins were manually removed during various intercultural operations, contributing to the control efforts against pomegranate wilt.

#### 3.4.2 Efficacy of fluopyram (34.48 %, SC) and fluensulfone (2 %, GR) against larval population in soil and galling index on pomegranate roots under pot conditions

Two consecutive experiments were conducted during the year 2019 and 2021 on pomegranate plants growing in plastic pots. During each experiment J2 larval suspension was poured twice at 15 days interval at a rate of 1000 larvae/pot (using a 10 ml nematode suspension) and experiment were laid out completely randomized block design (CRD) where each treatment were replicated seven times. Adequate moisture in the pot soil was maintained through frequent irrigation and soil samples were collected from each replicate at the establishment of disease symptoms (partial wilting) to determine the population of second-stage juvenile (J2) larvae presents in individual pots. Following this, 1 L solution of Fluopyram (34.48 %, SC) and Fluensulfone (2 %, GR) nematicides at a rate of 2 g/ml per liter, were drenched twice after 15 days interval and a separate control treatment were maintained without nematicides drenching. After 30 days from the second application of nematicides, soil samples were taken again from the treated and untreated pots and the population of J2 larvae was counted under a stereoscopic microscope at 40× magnification. Galling severity on roots was examined by observing the galling index for each treatment before and after application of nematicides during collection of soil samples.

### 3.5 Efficacy of fluopyram (34.48 %, SC) and fluensulfone (2 %, GR) against larval population in soil and galling index on pomegranate plant roots under field conditions

Field evaluation of Fluopyram (34.48 %, SC) and Fluensulfone (2 %, GR) were conducted during the year 2019 and 2021 in a pomegranate orchard located at the Modal Farm of the university. During each year in June month the plants (partially wilted) were selected based on wilting and galling symptoms and soil samples were collected from each treatment to count the population of second-stage juvenile (J2) larvae under a stereoscopic microscope before the drenching of the nematicides. Each treatment was drenched twice at interval of 15 days @ 8–15 L nematicides solution at a concentration of 2 g or ml/L according to size and vigour of the plant and a separate control treatment were maintained without nematicides drenching on it. Experiment was conducted using randomized block design (RBD) and seven replications were maintained for each treatment. After 30 days of second drenching soil sample was collected again for J2 larval population count in treated and untreated soil. The infested root samples from each treatment to access galling index were taken with soil samples before and after drenching by using scale mentioned earlier.

### 3.6 Statistical analysis

The statistical analysis was carried out using Rstudio and the dataset was imported into R software using csv. file.

#### 3.6.1 Surveillance of Kullu, Mandi and Solan districts

This study delves into the dynamic patterns of J2 larva per 100 cc soil and galling index across diverse locations within three districts. The comprehensive statistics unveils significant insights into the distribution and variability of these variables. This serves as a foundational step towards unraveling their interrelationships and ecological dynamics. The investigation includes the exploration of the correlation between J2 larva per 100 cc soil and galling index across the years 2018, 2019 and 2021. Through a correlation matrix, the study elucidates the strength and direction of connections between these variables, providing valuable insights into their associations. Furthermore, the distributional characteristics of J2 larva per 100 cc soil and galling index across various locations and years are visually depicted using box plots which offer a lucid portrayal of the data's variability and central tendencies, enhancing our understanding of the studied phenomena.

#### 3.6.2 Evaluation of nematicides under pot and field conditions

The data on galling index and J2 larval population before and after drenching of nematicides were compared using paired *t*-test to find out the significant difference in the means of J2 larval population (t(df) = [t-value], p = [p-value]). The overall significance of the MANOVA model was assessed using the multivariate F-statistic (F(df1, df2) = [F-value], p = [p-value]) to analyze multiple dependent variables simultaneously, including J2 larval population and galling index to find out significant differences in the combined dependent variables before and after drenching of nematicides. The box plot serves as a visual aid to understand the impact of drenching nematicides on the J2 larval population and offers an intuitive means to interpret the result of the study. Within the plot, each box depicts the inter-quartile range (IQR) of J2 larval population counts corresponding to the "Before" and "After" time points. Additionally, the median value is represented by a line within each box. The "whiskers" extend to 1.5 times the IQR from the lower and upper quartiles, encapsulating the minimum and maximum values. Comparing the box plots for the "Before" and "After" conditions enables a visual assessment of any shifts in the distribution of J2 larval population counts.

## 4 Results and discussion

### 4.1 Survey

Table 1 presents data on the occurrence of pomegranate wilt disease caused by *M. incognita* in Kullu, Mandi and Solan districts, as well as the distribution of second-stage juvenile (J2) larvae in the infested soil. Common symptom observed were yellowing of leaves (partial wilting) and reduction in size of leaves as compared to healthy plant and the infested plant remains stunted which was commonly observed in patches within an orchard. Nauni (Solan) exhibited the highest average nematode infestation (9.25 %) during the years 2018, 2019, and 2021 followed by Hurla (Kullu) with a rate of 7.42 percent (Fig. 2) while the lowest average disease incidence (0.36 percent) was recorded at Shilli (Solan). Galling index was noticed high in severely infested pomegranate plant among the surveyed locations (Fig. 3), Takoli had the highest galling index at 3.07 followed by Nauni at 2.94 (Table 1) while lowest galling index of 0.66 was observed at Shilli (Solan). The population of J2larva was observed higher in severely infested areas and average population ranged from 0 to 559 larvae/100ccsoil in Solan district, 0 to 547 larvae/100 cc soil in Mandi district and 125 to 580 larvae/100 cc soil in Kullu district during year 2018, 2019 and 2021 (Fig. 4).

**Table 1:** Occurrence of *M. incognita* incited pomegranate wilt disease in Kullu, Mandi and Solan districts and distribution of J2 larva in soil during year 2018, 2019 and 2021.

Disease incidence (%)						Galling index				J2 population (larva/100 cc soil)			
**Districts**	**Location**	2018	2019	2021	Avg.	2018	2019	2021	Avg.	2018	2019	2021	Avg.
**Kullu**	**Bajaura**	11.11	4.44	6.11	7.22	3.66	2.11	2.77	2.84	580	261	352	398
	**Seobagh**	4.87	5.55	3.33	4.58	3.11	3.33	1.44	2.62	321	296	189	268
	**Hurla**	10.55	8.77	2.96	7.42	2.55	2.33	3.33	2.73	316	268	424	336
	**Gadsa**	2.22	8.88	10.00	7.03	1.11	3.22	2.44	2.25	125	409	329	287
**Mandi**	**Nagwain**	10.00	3.33	3.33	5.55	3.77	1.55	2.11	2.47	547	186	250	328
	**Takoli**	5.55	7.22	6.11	6.29	2.33	3.66	3.22	3.07	331	525	490	449
	**Panarsa**	7.22	3.33	3.88	4.81	2.33	1.33	1.88	1.84	386	185	248	273
	**Jhiri**	2.22	2.77	1.48	2.15	0.00	1.66	1.11	0.92	0	213	116	110
**Solan**	**Nauni**	10.55	6.66	10.55	9.25	3.50	2.55	2.77	2.94	559	341	436	445
	**Dharja**	6.11	2.22	0.00	2.77	3.33	1.33	0.00	1.55	396	162	0	186
	**Shilli**	0.00	0.55	0.55	0.36	0.00	1.00	1.00	0.66	0	80	120	66
	**Kandaghat**	4.44	3.33	5.55	4.44	1.88	1.44	3.33	2.21	278	207	411	299

**Fig. 1:**
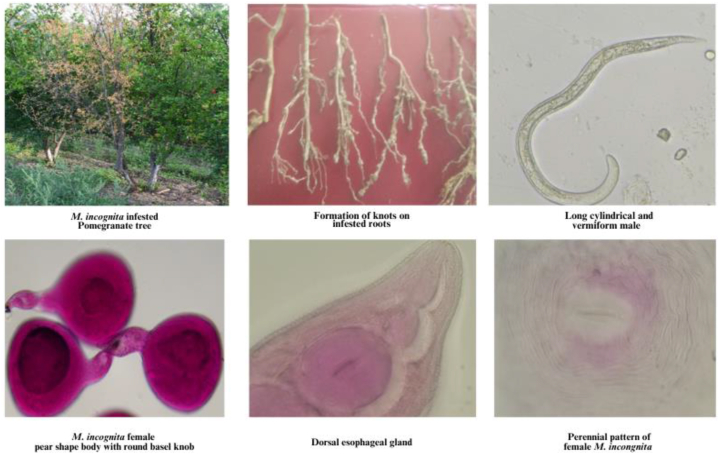
Symptomatology and Etiology of Pomegranate wilt disease caused root knot nematode.

**Fig. 2:**
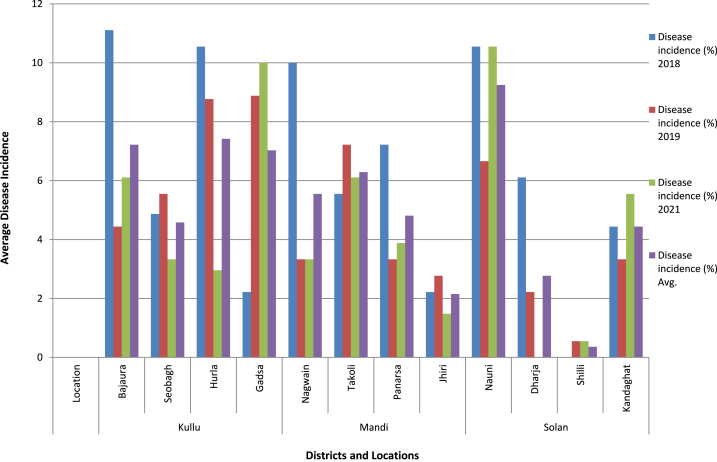
Occurrence of pomegranate wilt disease due to M. incognita in Kullu, Mandi and Solan districts during 2018, 2019 and 2021.

**Fig. 3:**
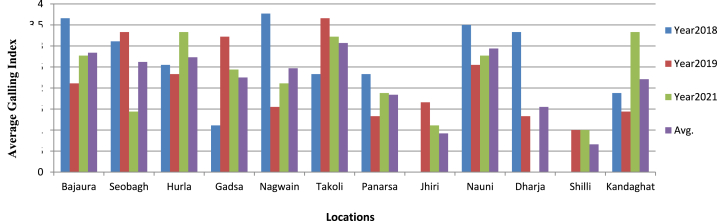
Average galling index on wilt infested pomegranate plant in Kullu, Mandi and Solan districts.

**Fig. 4:**
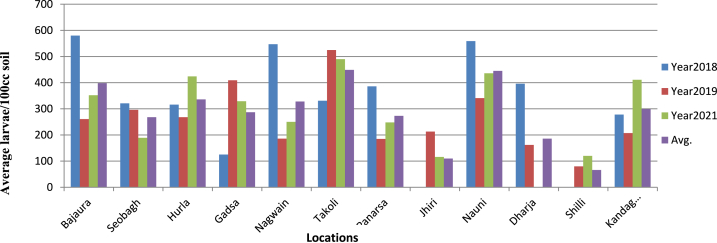
Larval population in wilt infested pomegranate plant basin soil in Kullu, Mandi and Solan districts.

**Fig. 5:**
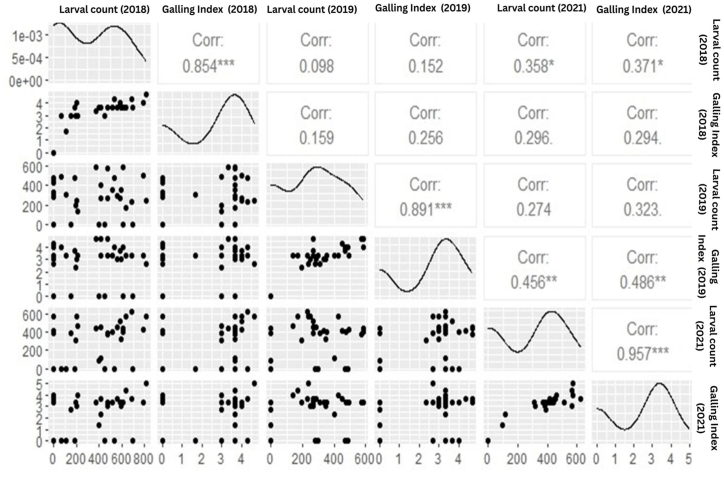
Correlation matrix of Average J2 population (larva/100 cc soil) and galling index across all three districts.

Spatial variability is evident from discrepancies in summary statistics across locations, while temporal variability is discernible from fluctuations in nematode populations and galling severity over time (Table 2). The statistical analysis elucidates the distribution of each variable across various locations and the median value for Galling Index (2018) was observed 3.67 indicating that 50 % of the locations exhibited a galling index of 3.670 or lower in 2018. Average value for the galling index across all locations in 2021 was recorded as 2.333. The maximum value observed for each variable across all locations in 2019 was 626.0 (Table 3). Correlation coefficients, ranging from −1 to 1 which offers a valuable insights into the relationships between variable and a strong positive correlation of 0.8539 exists between J2 larva/100 cc.soil and Galling Index (2018)," suggesting a simultaneous increase in both variables (Fig. 5). Similarly, Galling Index (2019) and Galling Index (2021) exhibited a strong positive correlation of 0.9565 (Table 4) indicating parallel trends in their fluctuations underscores positive associations between larva count and galling index values, their varying strengths across different years indicating a potential relationship between nematode population levels and plant damage as higher larva counts may correspond to increased galling severity. Furthermore, the summary statistics and correlation analysis collectively reveal spatial and temporal variability in larva counts and galling index values.

**Table 2:** Average galling index and J2 larval population/100 cc soil at different orchard during all three year surveyed.

Location	2018 J2 larva/100 cc soil (Average)	2018 Average Galling Index	2019 J2 larva/100 cc soil (Average)	2019 Average Galling Index	2021 J2 larva/100 cc soil (Average)	2021 Average Galling Index
**Bajaura 1**	785.33	4.0	496.67	3.33	422.67	3.33
**Bajaura 2**	560.67	3.67	288.33	3.0	454.67	3.67
**Bajaura 3**	396.67	3.67	0.0	0.0	94.67	1.33
**Seobagh 1**	105.0	1.67	300.0	3.33	0.0	0.0
**Seobagh 2**	210.67	3.0	136.67	3.33	462.67	4.0
**Seobagh 3**	636.0	3.67	167.67	3.33	568.0	4.33
**Hurla 1**	595.0	3.67	348.0	3.0	316.67	3.0
**Hurla 2**	0.0	0.0	473.33	4.0	399.67	3.33
**Hurla 3**	688.67	4.33	233.33	3.33	626.0	3.67
**Gadsa 1**	0.0	0.0	425.67	3.33	572.67	4.0
**Gadsa 2**	0.0	0.0	333.0	3.0	415.67	3.33
**Gadsa 3**	186.67	3.67	468.33	3.67	0.0	0.0
**Nagwain 1**	476.33	3.67	574.67	4.67	370.0	3.33
**Nagwain 2**	473.33	3.67	265.0	3.33	381.67	3.0
**Nagwain 3**	693.67	3.67	0.0	0.0	0.0	0.0
**Takoli 1**	581.33	4.0	266.67	3.67	513.33	3.33
**Takoli 2**	206.67	4.0	246.67	3.0	566.67	3.0
**Takoli 3**	413.33	3.67	266.67	4.67	451.33	3.67
**Panarsa 1**	150.0	3.0	0.0	0.0	390.0	2.67
**Panarsa 2**	606.67	3.67	586.67	4.0	396.67	3.33
**Panarsa 3**	518.33	3.67	354.67	3.33	399.67	3.33
**Jhiri 1**	0.0	0.0	308.33	2.67	384.67	3.33
**Jhiri 2**	0.0	0.0	281.0	2.67	0.0	0.0
**Jhiri 3**	0.0	0.0	0.0	0.0	0.0	0.0
**Nauni 1**	813.33	4.67	238.33	2.67	570.0	5.0
**Nauni 2**	190.0	3.0	194.67	2.33	310.0	3.33
**Nauni 3**	610.0	3.67	0.0	0.0	443.33	3.33
**Darja 1**	70.0	3.0	486.67	4.0	0.0	0.0
**Darja 2**	530.0	4.33	471.67	4.0	0.0	0.0
**Darja 3**	450.0	3.0	0.0	0.0	0.0	0.0
**Shili 1**	0.0	0.0	0.0	0.0	0.0	0.0
**Shili 2**	0.0	0.0	0.0	0.0	0.0	0.0
**Shili 3**	0.0	0.0	0.0	0.0	0.0	0.0
**Kandaghat 1**	370.0	3.33	586.67	4.67	442.0	3.33
**Kandaghat 2**	0.0	0.0	453.33	4.33	415.0	3.67
**Kandaghat 3**	415.0	3.67	400.0	3.33	113.33	2.33

1, 2 and 3 denotes orchard 1, 2 and 3 at each location.

**Table 3:** Summary statistics of variables across locations and years.

Nematode Population Density	J2.larva/100 cc.soil (2018)	Galling Index (2018)	J2.larva/100 cc.soil. (2019)	Galling Index (2019)	J2.larva/100 cc.soil. (2021)	Galling Index (2021)
1st Qu.	0.0	0.000	102.5	1.748	0.0	0.000
Median	383.3	3.670	273.8	3.330	387.3	3.330
Mean	325.9	2.584	268.1	2.611	291.1	2.333
3rd Qu.	565.8	3.670	432.6	3.670	445.3	3.330
Max.	813.3	4.670	586.7	4.670	626.0	5.000

**Table 4:** Correlation matrix of variables X2018 to X2021.

Variables	J2.larva/100 cc.soil (2018)	Galling Index (2018)	J2.larva/100 cc.soil. (2019)	Galling Index (2019)	J2.larva/100 cc.soil. (2021)	Galling Index (2021)
J2.larva/100 cc.soil (2018)	1.00000000	0.85396584	0.09847702	0.15230257	0.35764681	0.37144395
Galling Index (2018)	0.85396584	1.00000000	0.15892319	0.2561919	0.2962114	0.2943511
J2.larva/100 cc.soil (2019)	0.09847702	0.15892319	1.00000000	0.89138404	0.27403894	0.32251835
Galling Index (2019)	0.15230257	0.2561919	0.89138404	1.00000000	0.4560636	0.4855463
J2.larva/100 cc.soil (2021)	0.35764681	0.2962114	0.27403894	0.4560636	1.00000000	0.9565043
Galling Index (2021)	0.37144395	0.2943511	0.32251835	0.4855463	0.9565043	1.0000000

Previous studies by various researchers [10,[31], [32], [33]] have documented the presence of *M. incognita* infection on pomegranate plants. The wilt disease was initially observed in 2005 on pomegranate plantations in the valley areas of Kullu and adjoining Mandi district in Himachal Pradesh, as reported [34]. Subsequent studies [35,36] confirmed the ongoing presence and spread of the disease in the region. Furthermore, observations were made on the weed species present in the infected plant basins enlisted in Table 2 and weed species such as *Chenopodium album* Lin, *Solanum ptychanthum*, *Solanum nigrum, Amaranthus spinosus* and *Amaranthus viridis* indicates infestation by the root knot nematode *(M. incognita*). These weed species are likely contributing to the increased presence of the nematode in the infested soil. *M. incognita* infection on weed plants including *Amaranthus* sp. [37], *Solanum nigrum* and *Chenopodium album* has been previously reported by various researchers [38,39].

### 4.2 Symptomatology

The most common symptom observed on infested pomegranate plants affected by *M. incognita* was partial wilting, which led to a gradual decline in plant health and reduces the quantity and quality of produce. The infection was initially noticed as yellowing of leaves without drooping or partial wilting, (Fig. 1). On severe infection the flower, fruit leaves and branches of infested plant were observed reduced in size and plant growth were observed stunted as compared to healthy plant. Nematode galling or knots was prominent on the feeder root system on infested plant, particularly at a depth of 5–25 cm and initially appeared small but increased in size over time (Fig. 1). Galling on roots was observed as the abnormal growths or swellings that develop on infested plant roots in response *M. incognita* is characterized by the formation of localized swellings or tumors along the roots. Plants infested solely with *M. incognita* did not show immediate or significant decline. However, a slow decline in plant health was observed and drying of foliar part was observed after long term infection (Fig. 1). The findings on leaf yellowing, stunted growth and gall (knot) formation on the roots symptoms on pomegranate plant align with various previous studies [10,[30], [31], [32],^40^].

### 4.3 Identification of nematode

Through microscopic observation, it was determined that the mature female of *M. incognita* displayed a pear-shaped body with a rounded basal knob (Fig. 1) and the J2 larvae of *M. incognita* exhibited a vermiform (worm-like) shape (Fig. 1) and the average data on their metamorphic characters is mentioned in Table 6. The average length from dorsal esophageal gland orifice to base of stylet (DGO) was 3.56 μm (Fig. 1). This pattern is formed by the cuticle of the female's posterior end and includes features such as the stunted tail terminus, phasmids, lateral lines, vulva and anus surrounded by cuticular striae. The perineum of the female nematodes appeared oval to rounded, possessing a distinct dorsal arch with wavy striae and the lateral field was observed either absent or weakly demarcated (Fig. 1) during the examination. Similar observations regarding the pear-shaped body with a rounded basal knob of female *M. incognita* have been reported by previous researchers [30,41,42]. The findings from the morphometric analysis were consistent with previous study [43] who also reported average measurements for J2 larvae, including length (L) of 350 μm, stylet length of 11.50 μm, dorsal esophageal gland orifice to base of stylet (DGO) of 2.4 μm and tail length of 40.51 μm. Additionally, the measurements for *M. incognita* females were recorded as follows: length of 645.53 μm, stylet length of 14.20 μm and DGO of 3.6 μm.

**Table 5:** lists of weed plant species observed in wilt infested pomegranate plant basin in Himachal Pradesh and observation of root knot nematodes infestation.

Sr. No.	Botanical Name	Family	Common name	RKN association
1	*Solanum nigrum*	Solanaceae	Black Nightshade	Yes
2	*Solanum ptychanthum*	Solanaceae	Eastern Black Nightshade	Yes
3	*Physalis minima*	Solanaceae	Small-flowered Ground Cherry	Yes
4	*Datura stramonium*	Solanaceae	Jimsonweed	Yes
6	*Ageratum conyzoides*	Asteraceae	Billygoat Weed	No
7	*Acanthospermum hispidum*	Asteraceae	Bristly Starbur	No
8	*Parthenium hysterophorus*	Asteraceae	Congress Grass	No
9	*Sonchus*spp.	Asteraceae	Sowthistle	No
10	*Conyza*spp.	Asteraceae	Fleabane	No
11	*Cirsium arvense*	Asteraceae	Canada Thistle	No
12	*Galinsoga parviflora*	Asteraceae	Galinsoga	No
13	*Bidens pilosa*	Asteraceae	Blackjack, Hairy Beggarticks	Yes
14	*Amaranthus viridis*	Amaranthaceae	Green Amaranth	Yes
15	*Chenopodium album*	Amaranthaceae	Lamb's Quarters	Yes
16	*Digitaria sanguinalis*	Poaceae	Crabgrass	No
17	*Cynodon dactylon*	Poaceae	Bermuda Grass	No
18	*Echinochloa crus-galli*	Poaceae	Barnyard Grass	No
19	*Cyperus rotundus*	Cyperaceae	Nutgrass	No
20	*Trifolium repens*	Fabaceae	White Clover	No
21	*Oxalis corniculata*	Oxalidaceae	Creeping Woodsorrel	No
22	*Convolvulus arvensis*	Convolvulaceae	Field Bindweed	No
23	*Tribulus terrestris*	Zygophyllaceae	Puncturevine	No
25	*Commelina benghalensis*	Commelinaceae	Tropical Spiderwort	No
26	*Rumex*spp	Polygonaceae	Dock	No
27	*Euphorbia hirta*	Euphorbiaceae	Asthma Plant	No
28	*Coronopus didymus*	Brassicaceae	Lesser Swinecress	No
29	*Bacopa monnieri*	Plantaginaceae	Brahmi	No

**Table 6:** Metamorphic observations of root knot nematode (*Meloidogyne incognita*) under laboratory condition.

Life stage	Body part	Average mean diameter
J2(Juvenile 2)	Body length	344.2 (280–375)μm
	Stylet length	11.02(10.2–11.9)μm
	Hyaline Tail	36.20(34–38)μm
	Dorsal Gland orifice	2.58(2.2–3.1)μm
Adult Female	Body length	61.48 (56–64.5)μm
	Dorsal Gland orifice	3.56 (3.5–3.7)μm
	Stylet length	14.52(13.8–15.1)μm

### 4.4 Pathogenicity

**Fig. 6:**
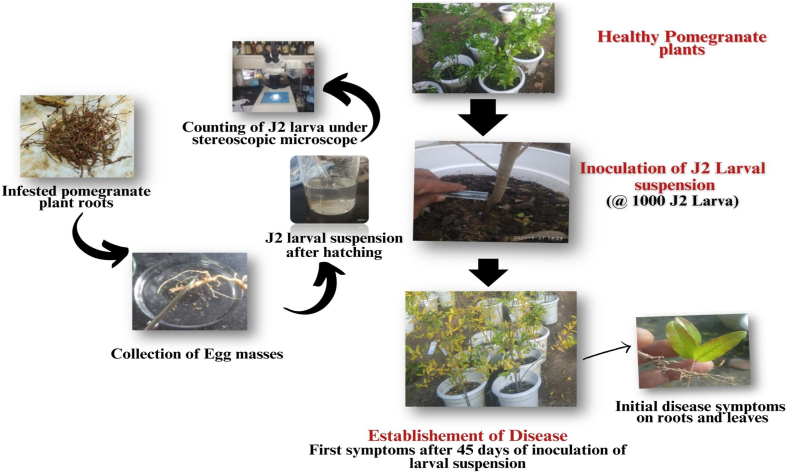
Pathogenicity test of *M. incognita* infection on pomegranate seedling under pot conditions.

Under laboratory conditions, hatching of egg masses was observed within an average time of 24–48 h and average 100 number of J2 larvae in 1 ml of the egg mass suspension was observed under stereoscopic microscope set at 40× magnification. The first symptom of yellowing leaves on one side of the pomegranate plant's canopy was observed 45 days after inoculation of larval suspension. Knot formation became noticeable after 60 days of larval inoculation and mature females were extracted from well-developed knots on pomegranate seedlings grown in pots after 90 days (Fig. 6). The control plants that were not inoculated the larval suspension and did not displayed any symptoms. Confirmation of *M. incognita* infection was achieved by observing similar characteristics, such as the oval to rounded shape of the mature female and the posterior cuticular pattern, which exhibited a high dorsal arch, usually wavy striae and a lateral field. Previous studies by researchers [31,41,42] have reported similar findings regarding the pear-shaped body and rounded basal knob of female *M. incognita.*

### 4.5 Management

#### 4.5.1 Cultural control

Implementing appropriate cultural practices is crucial for effective disease management and mitigating plant stress across various stages of crop growth [44]. Chemical drenching in poorly drained orchards proved ineffective in managing the disease while in orchards with adequate drainage combined with nematicides drenching, a successful reduction in both larval population and galling index on infested pomegranate plant roots was observed. Furthermore, integrating weed management strategies is essential for reducing nematode inoculum in the soil [45].The presence of weed species may facilitate continuous adaptation of *M. incognita* throughout the year. In this study, a total of 29 weed plant species from 14 different families were observed in association with wilted pomegranate plants in the districts of Kullu, Mandi, and Solan (Table 5). Among these, seven plant species belonging to three families (Solanaceae, Asteraceae, and Amaranthaceae) were found to be infested with root-knot nematodes, thus increasing their inoculum in the infested plant basin.

#### 4.5.2 Chemical control

##### 4.5.2.1 Pot experiments

**Fig. 7:**
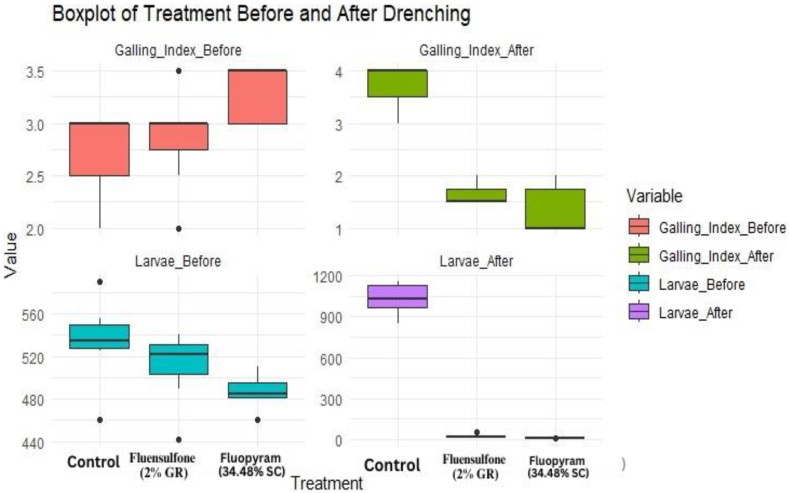
Box plots depicting the impact of nematicides drenching on J2 larval population and galling index under pot conditions.

The efficacy of Fluopyram (34.48 %, SC) was highly significant under pot condition with an average reduction of 99.00 percent J2 larva/100 cc soil of root knot nematodes after 30 days of 2nd drenching while Fluensulfone (2 %, GR) reduced 95.32 percent larval population of *M. incognita* (Table 7). In the control treatment without any chemical drenching, the population of *M. incognita* (J2 larvae) increased at a rate of 93.18 percent after 60 days of inoculations and the wilting symptoms gradually increased caused yellowing and stunting. Drenching of Fluopyram (34.48 %, SC) reduced the galling index on infected plant roots from 3.28 to 1.35 and drenching with Fluensulfone (2 %, GR) reduced galling index from 2.92 to 1.64 after 30th days after 2nd drenching while on control treatment galling index increased at the rate 36.84 percent from 2.71 to 3.71 (Fig. 7). These results suggest that the drenching treatment has efficacy in reducing both galling severity and nematode population density in the soil (Fig. 7). The minimum, 1st quartile, median, mean, 3rd quartile and maximum values of after drenching provide insights into the effectiveness of the drenching treatment in reducing root galling caused by *M. incognita* (Table 8). Lower values after drenching compared to before suggest a potential efficacy of the drenching treatment in controlling nematode infestation.

**Table 7:** Effect of Fluopyram (Pyridinyl-Ethyl-Benzamide) and Fluensulfone 2 % on J2 population of *M. incognita* in soil and galling index on pomegranate roots under pot conditions.

Treatment	Galling Index Before Drenching	Galling Index After Drenching	% Reduction in Galling Index	J2 Larvae/100 cc Soil Before Drenching	J2 Larvae/100 cc Soil After Drenching	% Reduction In Larval count
Fluopyrum	3.28	1.35	58.69	493.57	4.92	99.00
Flunosulphane	2.92	1.64	43.90	467.50	21.85	95.32
Control	2.71	3.71	36.84	534.28	1032.14	93.18

**Table 8:** Summary statistics of Galling Index and J2 Larvae Count before and after soil drenching.

Treatments	Galling Index (before drenching)	Galling Index (after drenching)	Larval population/100 cc soil (before drenching)	Larval population/100 cc soil (after drenching)
Min	2.0	1.0	440.0	5.0
1st Qu.	2.5	1.0	450.0	8.0
Median	3.0	1.5	455.0	50.0
Mean	3.0	2.048	485.2	359.4
3rd Qu.	3.5	3.5	495.0	880.0
Max.	4.0	4.0	652.5	1150.0

The null hypothesis states that there is no effect of treatment on the dependent variables (e.g., galling index, larvae count).The MANOVA results provide evidence that the treatment has a significant impact on the dependent variables collectively and the p-value (0.000663) was smaller than significance level (0.05) indicated strong evidence against the null hypothesis (Table 9). Therefore this study supporting the alternate hypothesis that there are differences among the treatment groups in terms of the variables under consideration. Therefore, it can be concluded that the treatment has a significant effect on the combined dependent variables, indicating that at least one of the treatment groups differs significantly from the others in terms of the multivariate response. The paired *t*-test results indicate a statistically significant difference suggesting that the treatments have a measurable effect on root damage severity as indicated by the Galling Index. The t-value (2.7755) represents the ratio of the difference between the means of the paired observations to the standard error of that difference which indicates the magnitude of the difference relative to the variability in the data (Table 10). The p-value associated with the *t*-test (0.01167) was less than the significance level (typically 0.05) suggests evidence against the null hypothesis. The alternative hypothesis states that the true mean difference between Galling Index before and after drenching treatment is not equal to zero. The 95 % confidence interval for the mean difference in Galling Index before and after drenching treatment is (0.2366074, 1.6681545). Fluopyram is known for its excellent safety profile function as a succinate dehydrogenase inhibitors (SDHI) nematicide. Its application has demonstrated effectiveness in rendering the population of *M. incognita* immotile [46]. Earlier studies [47] have shown that fluensulfone also has a harmful impact on the larval population of M. incognita. These findings are consistent with a study [48] which also observed a significant reduction in gall severity on *M. incognita*-infested *Cucumis sativus* roots after soil treatment with fluensulfone, fluazaindolizine, or fluopyram. The study also reported a significant difference (P < 0.0001) in nematode inoculation densities and the effectiveness of nematicides.

**Table 9:** Summary of MANOVA Results for Treatment Effects under pot condition.

	Df	Pillai	Approx F	num Df	den Df	Pr(>F)
Treatments	2	1.085	4.7432	8	32	0.000663
Residuals	18

**Table 10:** Paired *t*-test Results for Test Value Comparison (pot condition).

Test	Paired *t*-test (Value)
Sample Size (df)	20
t-value	2.7755
p-value	0.01167
Confidence Interval	(0.2366074, 1.6681545)
Mean Difference	0.952381
Alternative Hypothesis	True mean difference is not equal to 0

##### 4.5.2.2 Field evaluation

**Fig. 8:**
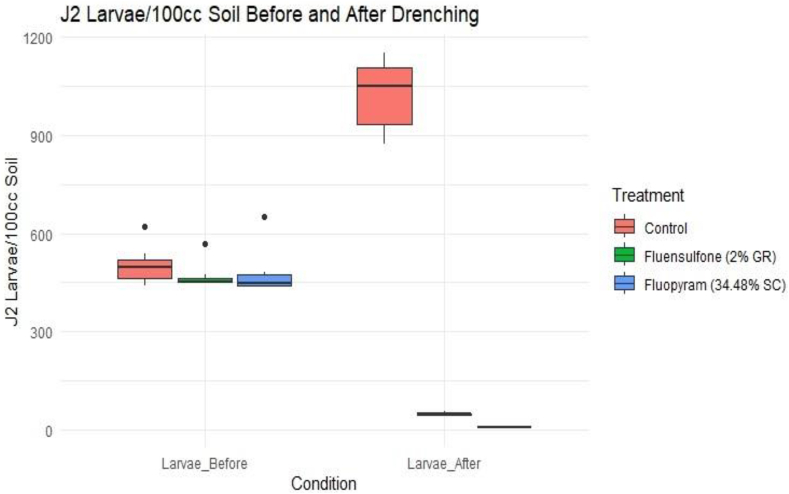
Box plot illustrating the variation in J2 larval population before and 30 days after the second drenching in the soil of infested plant basins.

**Fig. 9:**
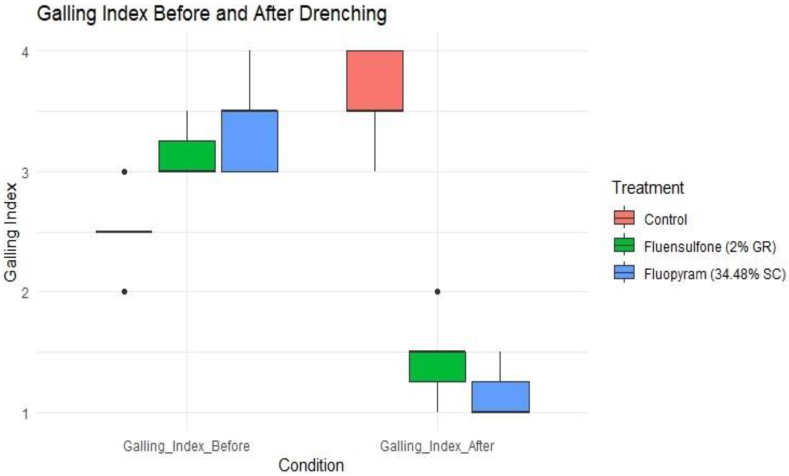
Box plot illustrating the impact of nematicides drenching on the galling index of infested pomegranate plant roots under field conditions.

Drenching the infested plants with Fluopyram (34.48 %, SC) at the rate of 2 ml/L was highly significant with average reduction of 98.56 percent larvae/100 cc in soil (30 days after 2nd drenching) while an average 89.32 percent reduction of J2 larva/100 cc soil was observed after Fluensulfone (2 %, GR) drenching (Fig. 8). In the control treatment without any chemical drenching, the population of *M. incognita* (J2 larvae) increased at a rate of 103.55 percent larvae/100 cc soil (Table 11) which causes gradual increase in plant wilting symptoms. Application of Fluopyram (34.48 %, SC) reduced the galling index from 3.35 to 1.14 accounting average 65.95 % reduction while Fluensulfone (2 %, GR) treatment reduced galling index from 3.14 to 1.42 with average 54.54 % reduction (30 days after 2nd drenching) (Fig. 9). The galling index on control treatment without any nematicides drenching increased at the rate of 45.71 percent from 2.50 to 3.64. The statistics provide insights into the distribution and central tendencies of the Galling Index and J2 larvae counts before and after the treatment and help in understanding the variability and effectiveness of the treatment (Table 12). There is a noticeable reduction in J2 larvae counts after the drenching treatment, as evidenced by the lower mean and median values of J2 larvae counts after the treatment compared to before. The observed change in the galling index before and after treatment exhibited a negative trend in treated plants, indicating a reduction in both larval population in the soil and galling index on infested roots. Conversely, the control treatment, which did not received chemical drenching, demonstrated a positive change with an increase in J2 larva and galling index, as depicted in Fig. 8, Fig. 9.

**Table 11:** Effect of Fluopyram (Pyridinyl-Ethyl-Benzamide) and Fluensulfone 2 % on J2 population of *M. incognita* in soil and galling index on pomegranate roots under field conditions.

Treatments	Galling Index Before Drenching	Galling Index After Drenching	% Reduction in Galling Index	J2 Larvae/100 cc Soil Before Drenching	J2 Larvae/100 cc Soil After Drenching	% Reduction In Larval count
Fluopyrum	3.35	1.14	65.95	498.57	7.142	98.56
Flunosulphane	3.14	1.42	54.54	457.85	48.85	89.32
Control	2.50	3.64	45.71	502.14	1022.14	103.55

**Table 12:** Summary statistics of Galling Index and J2 Larvae Count before and after soil drenching.

Treatments	Galling Index (before drenching)	Galling Index (after drenching)	Larval population/100 cc soil (before drenching)	Larval population/100 cc soil (after drenching)
Min	2.00	1.00	442.50	3.00
1st Quartile	3.00	1.50	485.00	6.00
Median	3.00	2.00	516.00	17.50
Mean	2.97	2.23	510.50	353.00
3rd Quartile	3.50	3.50	533.00	910.00
Max	3.50	4.00	590.00	1155.00

The MANOVA results indicate a significant overall effect of treatment on the combined dependent variables (galling index and larval population) with a Pillai's trace value of 1.4999 and a highly significant approximate F-statistic of 11.996 (p < 0.001) (Table 13) indicates a strong effect of the treatment on the dependent variables. This statistic tests the null hypothesis that there are no differences between treatment groups and the higher the F-value of 11.996 is highly significant (p < 0.001), indicating that the treatment has a significant effect on the combined dependent variables. The p-value indicates the probability of observing an F-value as extreme as the one obtained under the null hypothesis of no treatment effect. A p-value less than 0.05 (or any chosen significance level) suggests that the treatment has a significant effect on the dependent variables.

**Table 13:** MANOVA results for treatment effects.

	Df	Pillai	Approx F	Num Df	Den Df	Pr(>F)
Treatment	2	1.4999	11.996	8	32	1.014e-07 ***
Residuals	18					

The paired paired t-tests revealed that all three treatment groups (Control, Fluensulfone, and Fluopram) exhibits statistically significant differences between the measurements taken before and after the drenching treatment (Table 14). The p-values for the Control, Fluensulfone and Fluopram treatments were 0.0000950, 0.000433, and 0.0000281, respectively. The paired *t*-test for the Fluopram group produced the smallest p-value of 0.0000281 means the difference between the measurements before and after the Fluopram treatment is highly significant.

**Table 14:** Paired *t*-test Results for Treatment Groups under field conditions.

Treatment	Paired *t*-test
Control	0.0000950
Flunosulphane	0.000433
Fluopyrum	0.0000281

Comparing the results from both pot and field conditions provide valuable insights into the effectiveness of the treatments for managing *M. incognita* induced wilt disease in pomegranates plant. The treatments (Fluensulfone 2 % GR, and Fluopram 34.48 % SC) showed significant reductions in the galling index compared to the untreated condition and was supported by paired t-tests and MANOVA results, which indicated statistically significant differences in the galling index before and after treatment among the treatment groups. Similarly, under field conditions, the treatments (Fluensulfone 2 % GR, and Fluopram 34.48 % SC) also showed significant reductions in the galling index compared to the untreated condition. However, the field conditions introduce additional complexities, such as variations in environmental factors and soil conditions may influence the effectiveness of the treatments in the long term. Previous study [49] investigated the impact of fluopyram on the soil-dwelling second-stage juveniles (J2) and eggs of *M. incognita* and revealed a reduction in nematode population and a substantial increase in tomato plant yield, ranging from 21 % to 58 %. These results indicate the toxic nature of Fluopram 34.48 % SC towards *M. incognita*, highlighting its potential as an effective management tool against this plant parasitic nematode. The nematicides application reduced the J2 larval population in infested plant basin and reduced the galling index of the roots and ultimately leading change in foliar disease severity resulting successful management of the disease under field condition. Overall, the results from both pot and field conditions support the efficacy of the treatments in reducing the galling index and suggest their potential utility for managing *M. incognita* wilt disease in pomegranates.

## 5 Future research or field trials

To optimize Fluopyram's application and integrate it into broader nematode management strategies, future research and field trials should focus on conducting field trials across diverse geographical locations with varying soil types and climatic conditions will provide insights into the effectiveness of Fluopyram in different environments over multiple growing seasons. Additionally, research should investigate the optimal timing and dosage of Fluopyram application to maximize efficacy against *M. incognita* while minimizing environmental impact. Exploring the integration of Fluopyram with other nematode management practices, such use of organic amendments and biological control agents, will help develop comprehensive and sustainable nematode management strategies. Furthermore, studies should assess the impact of Fluopyram on soil microbial communities to ensure it does not disrupt beneficial organisms or soil health. Monitoring nematode populations for resistance development and conducting on-farm demonstration trials will help validate Fluopyram's efficacy under real-world conditions and promote its adoption among growers.

## 6 Limitations and challenges Encountered during the research

The COVID-19 pandemic posed significant challenges to our research on the dynamics of Meloidogyne incognita infestation in pomegranate orchards in Himachal Pradesh during year 2020. The pandemic highlighted the necessity for adaptive management strategies and robust contingency planning. The disruptions experienced particularly in surveillance, field and pot experiments, emphasized the importance of flexible pest management approaches. The complete lockdown resulted in laboratory closures for over six months and there was the additional danger to life posed by the pandemic. By acknowledging these limitations, we can better contextualize our findings and enhance the resilience and effectiveness of future research and management efforts in the face of unforeseen global challenges.

## 7 Conclusion

This study revealed the dynamics of J2 larvae and galling index distribution within pomegranate-growing districts (Kullu, Mandi and Solan) of Himachal Pradesh, emphasizing the importance of understanding their temporal and spatial patterns for effective pest management. Our findings reveal fluctuations in nematode populations over time and spatial heterogeneity across locations, influenced by various environmental factors and agricultural practices. Through rigorous experimentation and analysis, we have demonstrated significant treatment effects of chemical drenching on both the galling index and larval population of *M. incognita* infection on pomegranate crops. Specifically, our MANOVA analysis highlights the efficacy of chemical drenching treatments Fluopyram and Fluensulfone in mitigating *M. incognita* infestations, leading to improved plant health and productivity through reduced galling and larval population densities. Moreover, this study underscores weeds identified in the infested plant basin, which serve as alternative hosts for nematodes and exacerbate infestation levels in pomegranate crops. By implementing comprehensive weed management strategies encompassing cultural and mechanical approaches, growers can suppress weed growth, reduce nematode reservoirs and optimize pest control outcomes, fostering long-term resilience and productivity in pomegranate cultivation.Therefor, the present investigation contributes to the advancement of sustainable pest management practices in pomegranate cultivation by advocating holistic approaches that address both nematode and weed infestations.

## Compliance with ethical standard

All the ethical standard have been followed.

## Funding

This research was conducted through the Central Assistance Scheme of the Indian Council of Agricultural Research (ICAR). All funding was received through the Department of Plant Pathology, Dr. Y.S.P. University of Horticulture and Forestry, Nauni, Solan (Himachal Pradesh), India. No additional funding or grants were received.

## Data availability

The data supporting the findings of this research are available from the corresponding author upon request.

## CRediT authorship contribution statement

**Mukesh:** Writing – review & editing, Writing – original draft, Methodology, Investigation, Conceptualization. **Kishore Khosla:** Validation, Supervision, Conceptualization. **Satish K. Sharma:** Supervision, Conceptualization.

## Declaration of competing interest

The authors declare the following financial interests/personal relationships which may be considered as potential competing interests:The authors declare that they have no known competing financial interests or personal relationships that could have appeared to influence the work reported in this paper. If there are other authors, they declare that they have no known competing financial interests or personal relationships that could have appeared to influence the work reported in this paper.
